# Maternal consumption and perinatal exposure to non-nutritive sweeteners: should we be concerned?

**DOI:** 10.3389/fped.2023.1200990

**Published:** 2023-06-12

**Authors:** Francisca Concha, Verónica Sambra, Paola Cáceres, Sandra López-Arana, Bielka Carvajal, Martín Gotteland

**Affiliations:** ^1^ Department of Nutrition, Faculty of Medicine, University of Chile, Santiago, Chile; ^2^ Department of Women and Newborn’s Health Promotion, University of Chile, Santiago, Chile; ^3^ Institute of Nutrition and Food Technology (INTA), University of Chile, Santiago, Chile

**Keywords:** breast milk, amniotic fluid, non-communicable disease (NCD), neonatal exposure, sweet taste receptors, sucralose, acesulfame (ACE), steviol glucosides

## Abstract

The context for this review is the rapid increase in the use of non-nutritive sweeteners (NNSs) instead of sugar in foods and beverages, a situation so prevalent in some countries that consumers are finding it increasingly challenging to access foods without NNSs. The benefits of consuming NNSs on obesity and diabetes are now being questioned, and studies have shown that they may exert physiological activities, sometimes independently of sweet taste receptor stimulation. Few studies, limited mainly to North American and European countries, have described the consumption of NNSs by pregnant or lactating women and infants. Most focus on beverages rather than foods, but all agree that consumption levels have increased dramatically. Although some studies report a negative impact of NNSs on the risk of preterm birth, increased birth weight and decreased gestational age, the level of evidence is low. Several studies have also reported increased weight gain in infancy, associated with maternal NNS intake. Interestingly, several NNSs have been detected in amniotic fluid and breast milk, usually (but not always) at concentrations below their established detection limit in humans. Unfortunately, the impact of chronic exposure of the fetus/infant to low levels of multiple NNSs is unknown. In conclusion, there is a stark contrast between the galloping increase in the consumption of NNSs and the small number of studies evaluating their impact in at-risk groups such as pregnant and lactating women and infants. Clearly, more studies are needed, especially in Latin America and Asia, to fill these gaps and update recommendations.

## 1. Introduction

High sugar diets are associated with higher risk of chronic non-communicable diseases (NCD) in children and adults (1, 2). Such diets, during pregnancy and lactation, may also affect child's health, promoting the development of hyperinsulinemia, impaired glucose tolerance and adiposity (3, 4). To address these adverse effects, non-nutritive sweeteners (NNSs) has been promoted as an alternative to sugar (5, 6). These food additives provide a sweet taste without calories and are used to prevent weight gain or facilitate weight loss. Due to the sustained global increase in NCD, their use in foods has increased significantly worldwide in all age groups, including women of childbearing age (7, 8). Acesulfame K, aspartame, cyclamate, saccharin, sucralose, and steviol glycosides are the most widely used NNSs. They differ in sweetness (Table 1) and all, except stevia (extracted from *Stevia rebaudiana*) are produced by chemical synthesis. An Acceptable Daily Intake (ADI) is defined for each NNS, that determines the amount that can be ingested daily over a lifetime without appreciable health risk (Table 1) (5, 9). Epidemiological, clinical, and preclinical studies have questioned the use of NNSs, pointing out that these additives may not have the expected effects on NCD or, worse, may promote them. Consequently, there is currently a strong controversy regarding the widespread use of NNSs and their real impact on health (10–14). Although so far, the evidence against NNSs is not so robust, the importance of this debate and the need to clarify the effect of NNSs, alone or in combination, cannot be ignored, particularly in at-risk populations such as pregnant or breastfeeding women and children under 2 years of age.

**Table 1:** Characteristics of the most commonly used NNSs.

	Acesulfame K^+^	Aspartame	Saccharine	Sucralose	Steviol glucosides	Cyclamate Na^+^
Structure	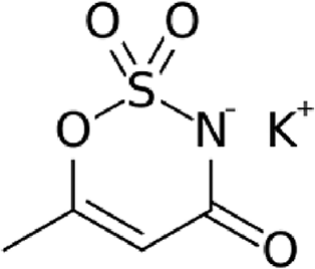	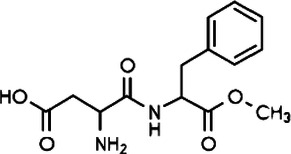	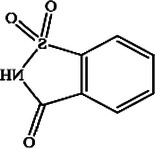	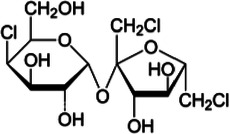	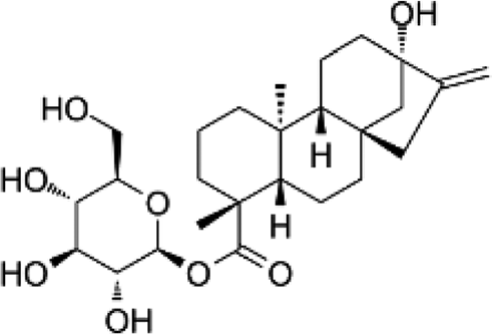	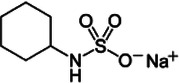
ADI (mg/kg)	15	40	5	15	4	7
Sweetness (% to sucrose)	160–220	180–200	∼300	∼600	150–300	30–50
Absorption/Metabolism	99% absorbed but not metabolized	Hydrolyzed by enterocyte brush border peptidases to Phe, Asp, methanol	Slowly absorbed and rapidly excreted. Not metabolized	15%–25% is absorbed and excreted in urine. About 7% could be stored in adipose tissue	Not absorbed. Deglycosylated by the colonic microbiota with release of steviol that can be absorbed	30% is absorbed and excreted in urine
Caloric value (kcal/g)	0	4	0	0	0	0

ADI, acceptable daily intake.

Therefore, the purpose of this narrative review is to provide an overview of the current evidence on perinatal NNS exposure and their potential impact on child health.

## 2. Physiological, toxicological and environmental aspects of the main NNSs

The absorption and metabolism of NNSs have been extensively studied (9). Aspartame is degraded by brush-border peptidases into phenylalanine, aspartic acid, and methanol. The latter can be oxidized to methanal and formic acid, which are toxic and could explain certain adverse effects reported with this NNS (15). Aspartame also reacts with chlorine in tap water, generating potentially toxic chloro-benzoquinone compounds (16). Regarding steviol glycosides, they are not absorbed in the intestine and reach the colon where they are degraded by the microbiota, releasing their aglycone fraction (steviol) which is absorbed and pass into the circulation as steviol glucuronide (17). With respect to the organochlorine sucralose, 20%/30% are absorbed in the intestine and are detected in urine up to 5 days after consumption (9). About 7% of this NNS is metabolized in the body, generating acetylated compounds that accumulate in adipose tissue, the physiological and health impact of such accumulation being unknown (18). Although frequently used in diabetic baked goods, high temperatures can degrade sucralose, producing chloropropanol and dioxins which are highly carcinogenic (19). Finally, sucralose is also used as a biomarker of gut permeability (20, 21). Most of acesulfame-K is absorbed in the intestine, but it is not metabolized or stored in the body and 99% is eliminated in the urine (9). Interestingly, sucralose and acesulfame K in wastewaters are resistant to the purification processes used in treatment plants. They are therefore not completely eliminated and, in the U.S., their presence has been detected in tap water (22). Accordingly, both NNSs are currently considered as new markers of anthropogenic pollution. Finally, NNSs also contribute to the dissemination of antibiotic resistance, favoring conjugative gene transfer between bacteria (23), which could be important both at the level of the individual (gut microbiota) and the environment.

## 3. Possible mechanisms involved in the adverse metabolic effects of NNSs

NNSs can impact the consumer's physiology through different mechanisms that, by themselves or synergistically, could explain some of the adverse metabolic effects generated by their consumption.

NNSs have a higher affinity than sugars for the sweet taste receptors (T1R1/T1R3) present on the taste buds of the tongue, and the enterocyte and enteroendocrine cell membranes. Stimulation of these receptors by NNSs increased the expression of the glucose-sodium co-transporter (SGLT1) and the insertion of the facilitated transporter GLUT2 into the enterocyte apical membrane, resulting in increased postprandial glucose absorption (24). Accordingly, obese subjects who underwent an oral glucose tolerance test had higher peak glycemia and insulin secretion if they had ingested sucralose before the test (25).

NNSs may also interfere with learned responses, contributing to glycemic control and energy homeostasis. Magnetic resonance imaging indicate that the activation of dopaminergic brain regions related to reward or pleasure is lower in subjects exposed to sucralose than in those exposed to sucrose, indicating that our brain differentiates between caloric and NNSs (26). NNSs would induce a dissociation between the sweet taste perceived in the mouth and the absence of calories associated with the food containing them, a phenomenon that could lead to a compensatory increase in appetite and energy intake (27, 28). Sucralose also tends to reproduce fasting in animals, by increasing the levels of AMP-kinase (an energy sensor) in nerve cells and stimulating the production of dopamine and neuropeptide-Y, increasing hunger sensation (29). NNSs, moreover, do not increase levels of plasma peptide YY and incretins involved in appetite and energy metabolism regulation (25, 30).

Finally, NNSs can affect the growth and/or metabolism of intestinal bacteria (31). They also promote glucose intolerance in mice, this phenomenon being prevented by antibiotics and transmissible to germ-free animals through fecal microbiota transplantation from exposed mice (32). The microbiota is therefore involved in the development of glucose intolerance induced by NNSs; similar observations were also reported in humans (32, 33). Interestingly, the microbiota of sucralose-treated animals is more virulent, contributing to liver inflammation, insulin resistance and adiposity (34).

## 4. Presence of NNSs in food and beverages: the example of Chile

The incorporation of NNSs in foods has rapidly increased worldwide in the last decade. In Chile, the implementation of the Nutrition Labeling Law 20.606, which imposes the application of warning labels on foods whose levels of critical nutrients are too high, has led the private sector to reformulate many foods and replace all or part of their sugar with NNSs (35, 36). In a recent study on 1,489 foods and beverages available in Santiago (8), we observed that 815 of them (55.5%) contained at least one NNSs, sucralose and steviol glycosides being the most frequent. This proportion was clearly higher than in other countries such as Brazil, Mexico, U.S.A., and Spain. Considering foods targeted more specifically to children, NNSs were present in 98.8% of juice powders, 98.3% of flavored milks, 91.2% of jellies, and 79% of dairy desserts. Chile is, therefore, among the countries in the world with the highest number of foods with NNSs, and for certain food categories, the consumer has almost no possibility to choose products without these additives. Such situation, which is also occuring in other countries, is a good illustration of the problem faced by nutrition and health professionals.

## 5. Maternal consumption of NNSs and health consequences

Recommendations on NNS consumption during pregnancy or in infants are variable and sometimes contradictory. In the U.S.A., while the Academy of Nutrition and Dietetics stated that NNSs (below ADI) was safe during pregnancy and infancy (37), the Institute of Medicine made no specific recommendation for pregnant women but cautioned against the use of NNSs in children, based on the lack of information about the eventual long-term adverse health effects of early exposure (38). These recommendations generally contrast to the real intake reported in young children (39). In Chile, the Diabetes and Pregnancy Guide of the Ministry of Health recommends a “moderate” use of aspartame, sucralose, acesulfame K and steviosides during pregnancy, and avoidance of saccharin (40), while in its Feeding Guide for children under 2 years of age (2015), it mentions that “Artificial sweeteners should not be used directly in foods for children under 2 years of age” (41). These recommendations were generally made when the use of NNSs in foods was not so frequent, compared to today.

A high proportion of women of childbearing age begin pregnancy overweight or obese and continue to consume NNSs during this period and during breastfeeding (42), to attenuate weight gain and the risk of gestational diabetes. Unfortunately, given the increasing number of foods with NNSs, it is likely that even women who do not want to consume them do so involuntarily. This situation is illustrated by an American study that detected the presence of sucralose in the urine of 44% of subjects who claimed not to consume NNSs (43). This unintentional consumption was attributed to the fact that certain foods were not labeled with sucralose content, and/or that this NNSs was also supplied by pharmaceutical products (syrups, electrolyte solutions, etc.), cosmetics (toothpaste), or possibly tap water (22). Unintentional consumption would also be favored by the fact that many parents were unable to correctly identify foods containing NNSs (44).

In fact, few studies have evaluated NNS consumption during pregnancy. In England, 5% of pregnant women followed in the context of the Born in Bradford cohort (*n* = 7,834) consumed drinks with NNSs (45), while they were 32.7% in the Danish National Birth Cohort (*n* = 59,334) (46), 63.4% in the Norwegian Mother/Child Cohort (*n* = 60,761) (47), and 29.5 in the CHILD cohort (*n* = 2,298) in Canada (48). In Chile, Fuentealba et al. reported that 98% of 601 women surveyed during pregnancy had used NNSs (42).

Whether NNSs are useful in reducing gestational diabetes is little studied and results are contradictory. While, in a cohort of 3,396 Spanish women, no relationship was observed between the consumption of diet drinks and the risk of gestational diabetes (as opposed to the consumption of sugar-sweetened beverages) (49), Nicoli et al. reported, in 376 pregnant mothers, that those who consumed drinks with NNSs had a higher risk of this condition compared to those who did not, even after adjusting the data for confounding variables (50). More studies are therefore necessary to improve our knowledge about the relation between NNS intake and this health concern.

Some studies have also assessed the effect of maternal consumption of NNSs on perinatal and early childhood risk factors. In the Danish cohort (46), pregnant women consuming NNS beverages had higher risk of preterm birth [adjusted OR: 1.46 (1.15–1.65) for >1 serving/day and 1.78 (1.19–2.66) for >4 servings/day, compared to <1 serving/day]. This association, found in both normal-weight and obese women, was not observed with consumption of sugar-sweetened beverages. Similar results were reported in Norway (47), but with no difference with mothers consuming sugar-sweetened beverages. Finally, Petherick et al. found no association between these variables (45). These different studies (*n* = 129,009) were subsequently pooled in a meta-analysis (51) that concluded that consumption of NNSs during pregnancy is associated with a significant 18% increased risk of preterm birth. Maternal NNS intake was also associated with a 24 g increase in birthweight and a significant decrease in gestational age (−0.11 weeks), but the authors considered the clinical significance of these changes questionable. Due to the degree of heterogeneity of the studies selected in the meta-analysis and the low quality of some of them, there was only a low level of evidence of a negative effect of NNS consumption during pregnancy on these perinatal parameters.

## 6. NNSs in breast milk and amniotic fluid

Some studies investigated the presence of NNSs in cord blood, amniotic fluid and breast milk to determine fetal/infant exposure to NNSs. In 2015, Sylvetsky et al. described for the first time the presence of saccharin, sucralose and acesulfame K in breast milk in 13 of 20 mothers (65%), including 4 who claimed not to consume them (52, 53). These authors also analyzed the pharmacokinetics of sucralose and acesulfame-K in 34 lactating women after ingestion of 58 and 41 mg of these NNSs, respectively (54). Sucralose and acesulfame K were detected in 21% and 18% of the milk samples before the NNSs were ingested. Acesulfame K appeared earlier (120 min) than sucralose (180 min) in breast milk, reaching concentrations between 4 and 7,388 ng/ml and between 299 and 4,764 ng/ml, respectively, without differences according the maternal nutritional status. These authors also described the presence of acesulfame-K and saccharin in 100% and 80% of 15 cord blood samples, with maximum concentrations of 6.5 and 2.7 ng/ml, respectively, while sucralose and steviol glucuronide were not detected (55). A similar study (56) in 49 lactating mothers after the ingestion of acesulfame-K, saccharin, cyclamate, and sucralose, detected their appearance in breast milk at 240–300 min, with maximum concentrations of 936, 81.5 and 2.56 ng/ml Again, these results were independent of the weight and metabolic status of the mothers. The presence of saccharin, acesulfame K, steviol glucuronide and/or sucralose has also been reported in 77% of amniotic fluid (*n* = 13) samples (55). The mean concentrations of these NNSs were 23, 10.7, 47.6, and 30.6 ng/ml for acesulfame K, saccharin, steviol glucuronide, and sucralose (only detected in one sample), respectively. Recently, our laboratory also detected the presence of NNSs in amniotic fluid and breast milk samples, at concentrations similar to those described above (unpublished data). Considering that amniotic fluid intake by the fetus is around 200–250 ml/kg per day (57), it can be calculated that a 1.000 g fetus (i.e., aged 26–27 weeks) could swallow approximately 4.60–5.75 μg of acesulfame K, 2.14–2.67 μg of saccharine, 6.12–7.65 μg of sucralose and 9.5–11.9 μg of steviol glucuronide, per day, amounts much lower than their corresponding ADI (if it makes sense to apply this concept to fetuses!!).

Globally, these observations confirm that NNSs may be transferred from the mothers to the fetus/infant through transplacental pathway and/or breast milk. This event could be favored by the fact that the gut permeability is physiologically increased in women during pregnancy and lactation (58, 59).

An important issue is whether the fetus/infant can detect NNSs at these concentrations in amniotic fluid and breast milk. As shown in Table 2, the concentrations reported in these studies are mostly below the detection taste threshold of each NNS (60). However, one subject showed outlier values for acesulfame K and sucralose in breastmilk (54) that, in the case of sucralose, exceeded its taste detection threshold. Although it is not clear why this mother had such high breastmilk concentrations of these NNSs, these results suggest that a small proportion of fetus/infants is chronically exposed to increased sweetness during gestation/lactation, a situation that could favor sweet taste receptor desensitization, increasing sweet taste threshold and affect food intake and weight gain during infancy. On the other hand, chronic exposure to NNSs is of concern, even if their concentrations are below their detection threshold. Indeed, NNSs can exert physiological activities independently of the stimulation of sweet taste receptor. Saccharin, for example, was shown to stimulate adipogenesis and inhibit lipolysis in this way (61), while sucralose lowered membrane order and reduced calcium flux in T-cells, affecting their response and function (62).

**Table 2:** NNS concentrations detected in amniotic fluid and breast milk samples, compared to detection taste threshold for each NNS.

NNSs	Detection taste threshold (μg/L) (60)	NNS concentrations in amniotic fluid (μg/L) (55)	NNS concentrations in breast milk (μg/L) (54)
Acesulfame K	8,940	23.0 ± 37.3	858 (∼4.500)^a^
Saccharin	2,690	10.7 ± 18.9	-
Sucralose	200–5,170	30.6	6.02 (∼7,400)^a^
Aspartame	6,590	-	-
Cyclamate	106,000	-	-
Sucrose	1,890,000	-	-

^a^ Extreme values reported in one subject by Rother et al. (54).

## 7. Impact of maternal intake of NNSs during the primary infancy

Few studies have investigated the effects of perinatal exposure to NNSs on the development of obesity in offspring. Azad et al. (63) followed 3,033 pregnant women and their children for 1 year. Twenty-nine percent consumed beverages with NNSs, 5.1% with a frequency of 1 serving per day. Offspring of mothers who consumed NNS beverages had a higher BMI z-score at 1 year and a 2-fold higher risk of being overweight than those from mothers who did not consume NNSs beverages. This effect was not explained by maternal BMI, diet quality, total energy intake or other risk factors for obesity. Consistent with these findings, a study in 918 Danish mothers with gestational diabetes showed that daily intake of NNSs during pregnancy was associated with a 0.59 standard deviation increase in the child's BMI *z*-score at 7 years of age, and a 1.9-fold increased risk of overweight/obesity (64). In both studies, the association were stronger in boys than in girls though the reasons for these differences were unknown. Plows et al. (65) followed 1,683 children to evaluate the impact of maternal NNS intake on BMI *z*-score and body fat during infancy. Compared with the first quartile of NNS intake, the highest quartile (0.98 ± 0.91 servings/day) was associated with higher BMI *z*-score in infancy (6.3 months), early childhood (3.2 years), mid-childhood (7.7 years), and early adolescence (12.9 years). Q4 was also associated with higher sum of skinfold in early childhood, mid-childhood, and early adolescence and higher fat mass index in mid-childhood. In contrast to these findings, Gillman et al., in 1,078 children born from mothers without gestational diabetes, observed no association between maternal intake of NNSs (expressed as a continuous variable) during pregnancy and increased adiposity during childhood (66).

Regarding the pediatric population, the National Health and Nutrition Examination and Surveys (NHANES) have reported a steady increase in NNS consumption in the United States. The percentage of children reporting consumption of foods or beverages with NNSs increased from 8.7% in 1999/2000 to 25.1% in 2009/2010 (67). In Chile, a recent study observed an increase of 40% in diet sweetness between 12 and 36 months of age (68). The main foods associated with sweetness were fruits and beverages (27.3% and 19.3% of total sweet density, respectively) at 12 months, and beverages and dairy products (32.2% and 28.6%) at 36 months. Fifteen percent of food items consumed by the children contained NNSs at 12 months of age and 14% at 36 months, the most common being sucralose (10% of food items), acesulfame (5%), and aspartame (4%). For results in older children, we refer to other studies (69, 70).

## 8. Conclusions and projections

The consumption of NNSs by pregnant/lactating women and young infants is little studied, while their use is increasing rapidly worldwide. Most of the available data come from North American and European countries and refer to beverages rather than foods. Few studies have assessed the effect of maternal consumption of NNSs on risk parameters associated with pregnancy and the nutritional status of infants during childhood. The fact that several NNSs can be found in amniotic fluid and breast milk, sometimes at concentrations above their detection limit, draws attention to the potential impact of this chronic exposure on the health of the fetus/infant. It would be interesting to study the interactions between the different NNSs in the body, beyond their individual effect, and to focus on the effects of NNSs produced through mechanisms independent of sweet taste receptors. There is therefore an urgent need for further studies in these risk groups, especially in Latin American and Asian countries, to fill the knowledge gap on NNSs and update the current recommendations.
